# Ultrasound-guided coracobrachialis plane musculocutaneous nerve block for perioperative analgesia in pediatric Gartland type III supracondylar humerus fracture: a prospective pilot study

**DOI:** 10.3389/fped.2025.1485277

**Published:** 2025-04-24

**Authors:** Tianyi Gao, Zhuorun Song, Shunyi Lu, Nan Song, Wentao Yu, Huilin Yang, Jun Zou, Qian Wang, Jun Ge

**Affiliations:** ^1^ Department of Anesthesia, Children’s Hospital of Soochow University, Suzhou, Jiangsu, China; ^2^ Department of Orthopedic Surgery, The First Affiliated Hospital of Soochow University, Suzhou, Jiangsu, China; ^3^ Department of Anesthesia, The First Affiliated Hospital of Soochow University, Suzhou, Jiangsu, China; ^4^ Department of Orthopedic Surgery, Children’s Hospital of Soochow University, Suzhou, Jiangsu, China

**Keywords:** coracobrachialis plane, musculocutaneous nerve block, pediatric supracondylar humerus fracture, perioperative analgesia, pain management, prospective pilot study

## Abstract

**Purpose:**

Perioperative pain management in children with Gartland Type III supracondylar humerus fractures (SHF) is crucial but often inadequately addressed, leading to significant pain experiences. This study aimed to evaluate the efficacy and safety of coracobrachialis plane musculocutaneous nerve block (Cora-MNB) compared to supraclavicular brachial plexus block (SC-BPB) for analgesia in pediatric Gartland Type III SHF patients.

**Methods:**

A prospective pilot study enrolled 105 pediatric patients with Gartland Type III SHF was performed. Primary outcome was the postoperative FLACC scale measured at 12 h postoperatively. Secondary outcomes included FLACC scale measured at 1 h, 6 h and 24 h postoperatively. They also included postoperative thumb and shoulder strength, opioid use, NSAIDs use, length of hospital stays, patient satisfaction, surgeon satisfaction, operation time and puncture channels. One hundred and five patients were randomized allocated between groups.

**Results:**

Patients receiving Cora-MNB showed superior analgesia, with median postoperative FLACC pain scores at 12 h reduced by 40% [Cora-MNB: 3.00 (2.00) vs. SC-BPB: 5.00 (2.00), ***p < 0.001]. Thumb extensor weakness incidence decreased significantly (Cora-MNB: 13.5% vs. SC-BPB: 84.9%, ***p < 0.001). Shoulder mobility preservation was achieved in 98.08% of Cora-MNB cases vs. 20.75% with SC-BPB (***p < 0.001). While opioid consumption showed no intergroup difference, Cora-MNB reduced NSAID rescue times [Cora-MNB: 0.00 (1.00) vs. SC-BPB: 1.00 (1.00), **p = 0.0014]. Procedure duration favored Cora-MNB [4.54 ± 1.21 (min) vs. 9.02 ± 1.94 (min), T = 14.32, 95% CI: 3.88–5.12, ***p < 0.001], with higher surgical and parental satisfaction scores. Hospital stays remained comparable [1.60 ± 0.66 (days) vs. 1.56 ± 0.67 (days), *p* = 0.98].

**Conclusion:**

Cora-MNB proves to be a safe and effective approach for anesthesia in pediatric SHF cases, offering superior analgesic outcomes, reduced NSAIDs usage, improved shoulder functionality, and high satisfaction levels without extending the hospital stay. This study supports the implementation of Cora-MNB as a valuable technique in perioperative pain management for pediatric SHF patients.

## 1 Introduction

Supracondylar humerus fracture (SHF) is the most common elbow fracture in the pediatric population (1, 2). Due to their benefits of causing less trauma and enabling faster recovery, closed reduction and fixation using percutaneous Kirschner (K)-wires have become the standard treatment for cases involving displacement and ulnar deformity (3, 4). However, perioperative pain management remains challenging, particularly in completely displaced Gartland Type III cases where most patients experience moderate-to-severe postoperative pain.

Current SHF analgesia predominantly relies on systemic opioids and brachial plexus blocks. However, opioids carry risks of postoperative nausea and vomiting (PONV) vertigo, and inadequate pain relief (5). Additionally, the overprescription of opioids for pediatric patients after the operation persists, further underscoring the urgent need for anesthesia optimization (6). While conventional brachial plexus blocks impair limb mobility and proprioception. The supraclavicular approach, though widely used, may provide excessive sensory blockade for distal fractures (7). Studies have been performed to optimize anesthesia and pain management for pediatric humerus surgeries. For example, Fan et al., demonstrated improved outcomes in terms of upper limb function and postoperative pain relief for children with lateral humeral condyle fractures through a combination of brachial plexus block and general anesthesia (8).

Anatomic studies reveal that the musculocutaneous nerve innervates the anterior periosteum of the distal humerus, a key pain generator in SHF (9). Conventional approaches to musculocutaneous nerve blockade through axillary brachial plexus blocks primarily target tourniquet pain rather than fracture-specific analgesia (10). Thus, the present study aims to optimize the anesthetic protocol for pediatric Gartland Type III SHF cases. Accordingly, we hypothesize that targeted ultrasound-guided musculocutaneous nerve block at the coracobrachialis plane could provide focused analgesia while preserving limb function.

## 2 Patients enrollment and methods

### 2.1 Sample size and study population

**Figure 1:**
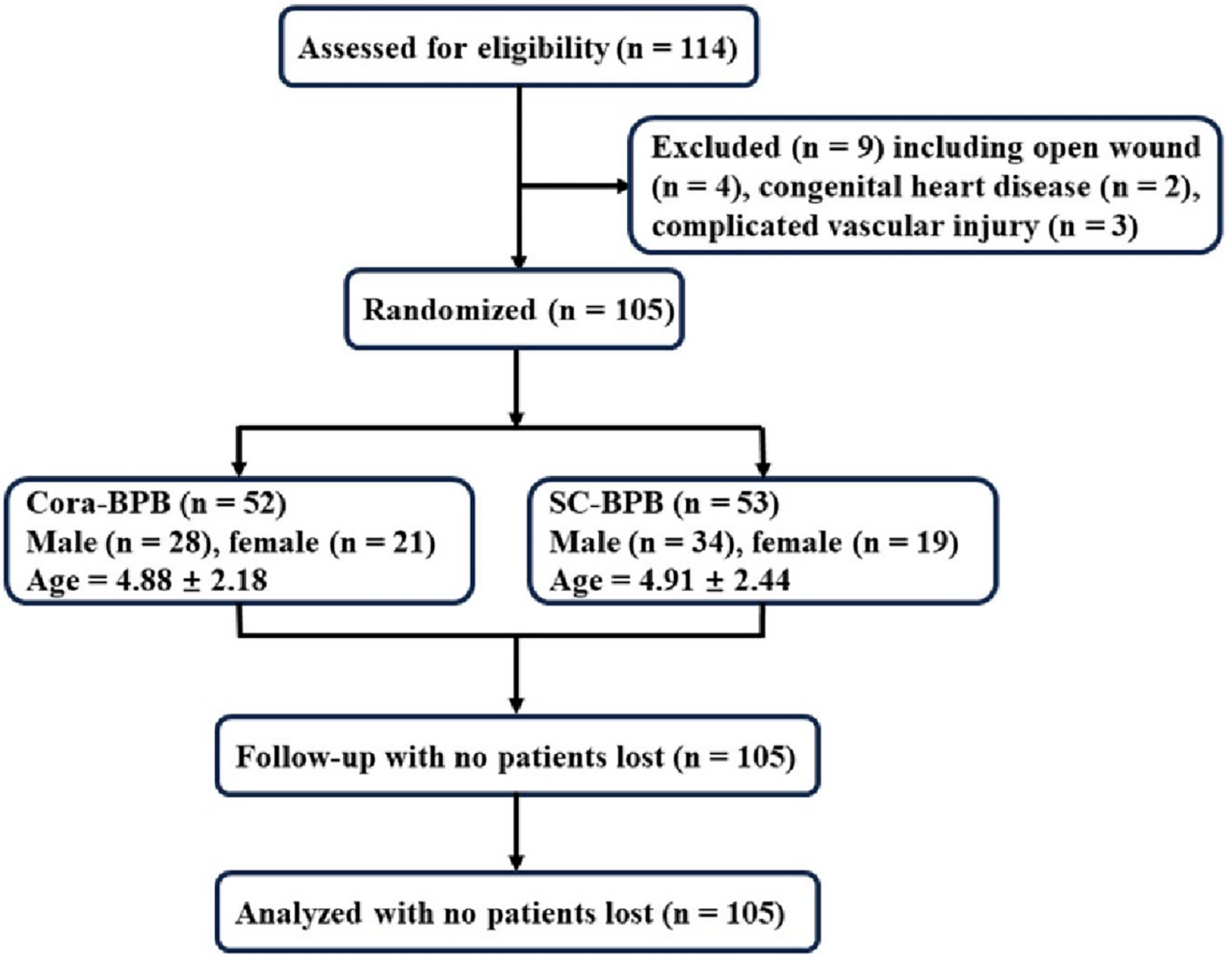
Consolidated standards of reporting trial flow diagram.

According to the previous work, at least 25 patients were required for each group to detect a 20% difference in primary outcome between the groups (11), assuming a power (1-β) of 80% and a two-sided type I error (α) of 0.05. Considering a 20% drop-out rate, at least 30 patients should be recruited in each group. Consolidated standards of reporting trial flow diagram could be found in Figure 1.

### 2.2 Exclusion criteria

(i) Patients with contraindications to anesthesia. (ii) Patients with a personal history of allergy to anesthetic agent. (iii) Patients with cardiovascular and cerebrovascular diseases (including but not limited to congenital heart disease, heart failure, coronary heart disease, cerebrovascular disease). (iv) Patients with respiratory insufficiency (including but not limited to bilateral rib fractures and obstructive emphysema). (v) Patients with abnormal coagulation function. (vi) Patients with open fracture and puncture site infection. (vii) Patients with a history of continuous use of analgesic drugs 3 months before surgery. (viii) Flexion-type SHF and SHF with vascular and nerve injury.

### 2.3 Randomized controlled implementation

Randomization was performed by an online randomization computer generator (https://www.sealedenvelope.com) on a 1:1 basis. The patients were randomly divided into two groups. The details of the group's assignment were placed into an envelope. Neither the patients nor the subsequent researchers (including the anesthetic nurse responsible for evaluating outcomes) knew the grouping information. The regional anesthesiologist opened the envelope after obtaining the patient's consent, and then performed the according anesthetic medication.

### 2.4 Grouping and anesthetic protocol

General anesthesia was firstly performed for the patients before regional anesthesia, beginning with intravenous induction using propofol at 3 mg/kg, fentanyl at 1 μg/kg, and rocuronium at 0.3 mg/kg. Subsequently, a laryngeal mask airway was inserted, secured appropriately, and inhalation of sevoflurane was initiated to maintain general anesthesia. Fentanyl was supplemented based on changes in heart rate and blood pressure. After general anesthesia, another experienced anesthesiologist offered regional anesthesia, under the supervision of another high-level anesthesiologist. All the regional anesthesia procedures were performed by the same anesthesiologists.

**Figure 2:**
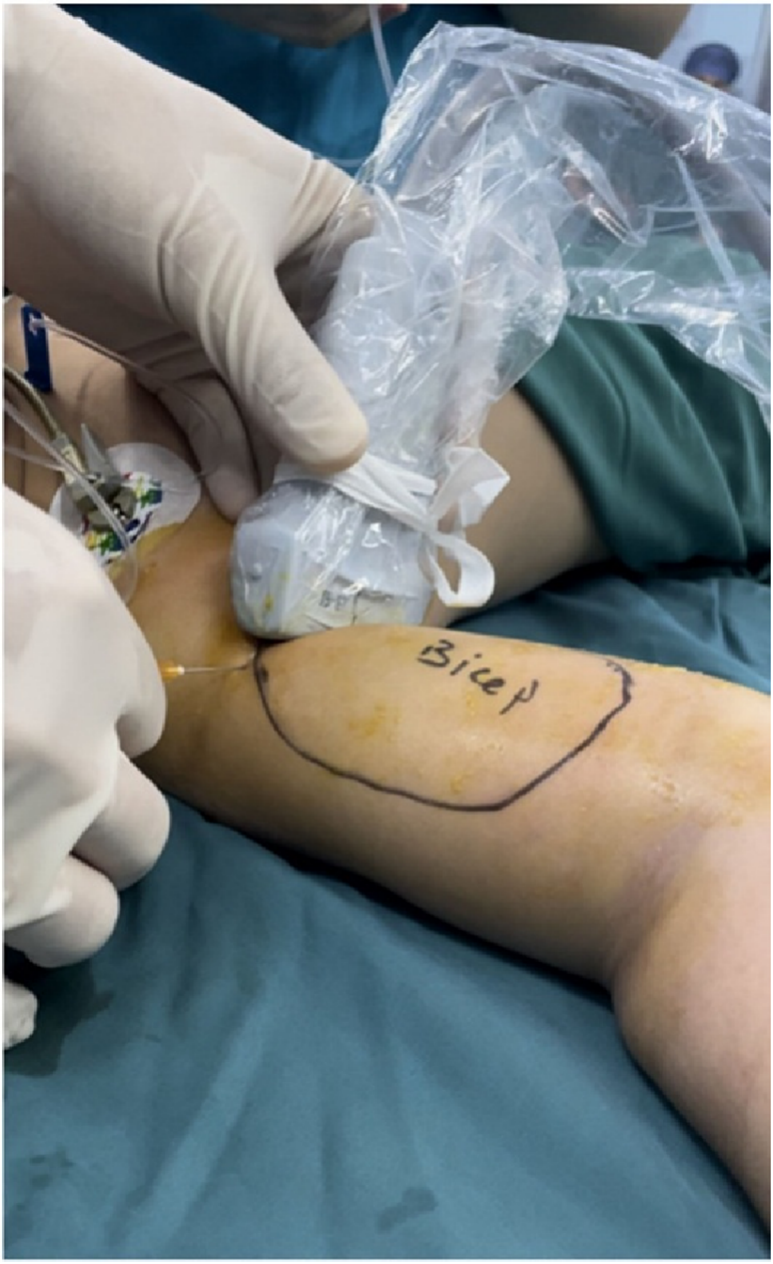
General image of ultrasound-guided coracobrachialis plane musculocutaneous nerve block.

**Figure 3:**
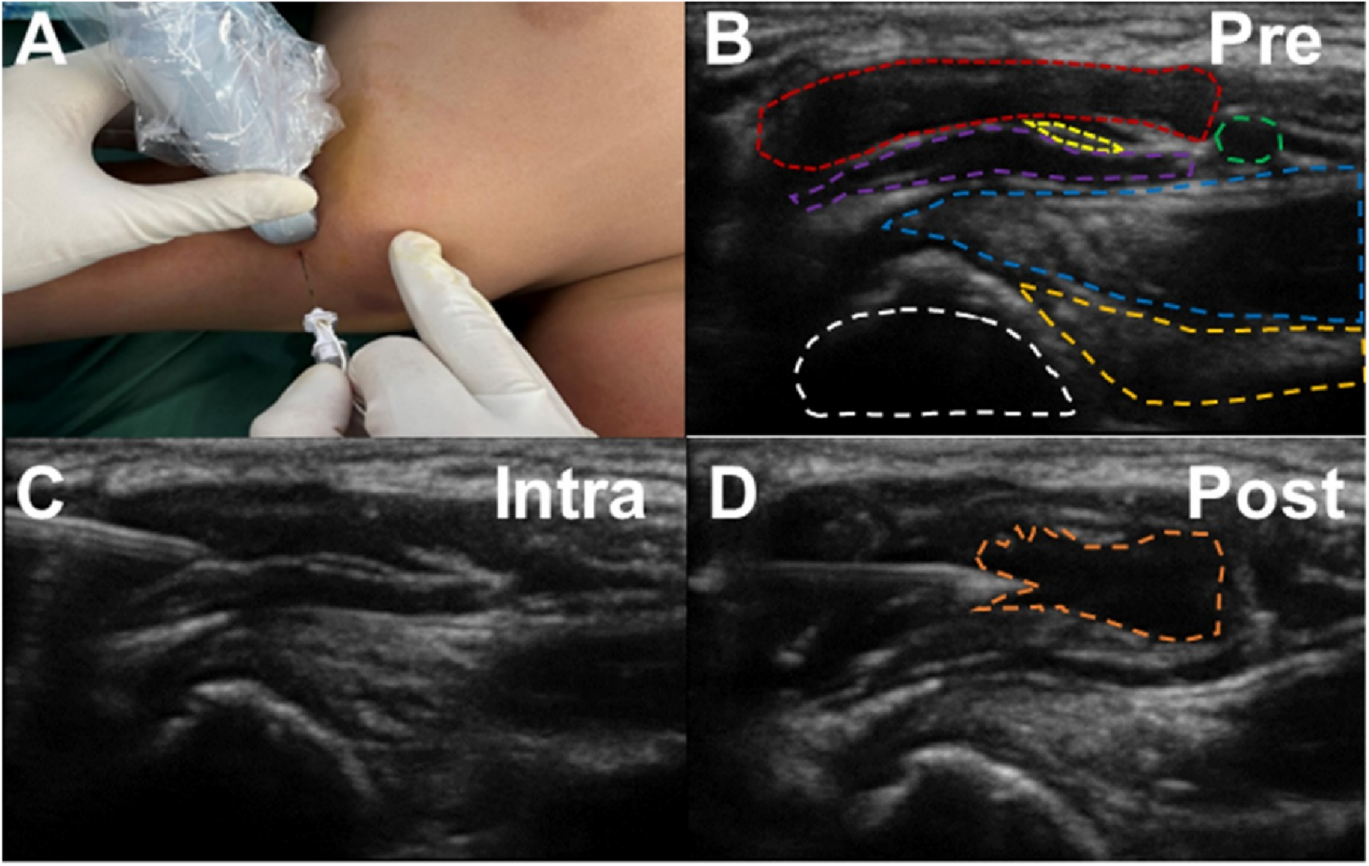
The representative patient's medical images of ultrasound-guided coracobrachialis plane musculocutaneous nerve block. **(A)** Pre-puncture ultrasound image. White: Humerus; Dark yellow: Teres major; Blue: Latissimus dorsi; Purple: Coracobrachialis; Yellow: Musculocutaneous nerve; Red: Biceps; Green: Axillary artery. **(B)** Intra-puncture ultrasound image: The puncture reaches the fascial layer. **(C)** Intra-puncture ultrasound image: The fascia is stretched by the injection. **(D)** Post-injection ultrasound image. Orange: local anesthetic agent injected.

For the first group of patients, we administered treatment through an ultrasound-guided coracobrachialis plane musculocutaneous nerve block. This group was designated as the Cora-MNB group. The coracobrachialis plane refers to the facial space located between the coracobrachialis and biceps muscles. The procedure was conducted under ultrasound guidance (Sonosite Mturbo HFL38x). Detailed ultrasound puncture images can be found in Figures 2, 3. The local anesthetic agent is 0.2% ropivacine. Injection volume (ml) is 0.4× body weight (kg).

For the second group, patients were treated with supraclavicular brachial plexus block. The group was named as SC-BPB. The procedure was also guided by ultrasound. The local anesthetic agent is 0.2% ropivacine. Injection volume (ml) is 0.4× body weight (kg). Local anesthetic concentration and dosage is based on the guidelines published by the New York School of Regional Anesthesia (NYSORA), specifically Peripheral Nerve Blocks for Children by Steve Roberts.

The type of surgical technique performed were left to the discretion of the treating physicians, following a local protocol that permitted slight variations. The study aimed to mirror real-world clinical practices, emphasizing high external validity.

After the operation, each patient received an intravenous injection of 0.1 mg/kg tropisetron. Subsequently, the patients were transferred to the Post-Anesthesia Care Unit (PACU), where the initial pain assessment was performed 1 h postoperatively. If the FLACC score exceeded 3, intravenous fentanyl was administered for pain relief. A reassessment was performed 10 min after medication administration, and patients were returned to the ward once the FLACC score was less than or equal to 3. Opioid analgesics (fentanyl) were used exclusively in the PACU for early postoperative pain control.

In the ward, oral NSAIDs served as the primary analgesic (ibuprofen suspension, 7 mg/kg/Time, Johnson&Johnson, USA), with supplementary doses capped at a maximum of 2 administrations per patient to ensure that the capping effect was not exceeded. Only in the rare event that a patient's pain remained inadequately controlled with oral NSAIDs (FLACC score >3) would the anesthesiologist, who administered the general anesthesia, rescue the situation with intravenous fentanyl.

### 2.5 Outcome measures

**Figure 4:**
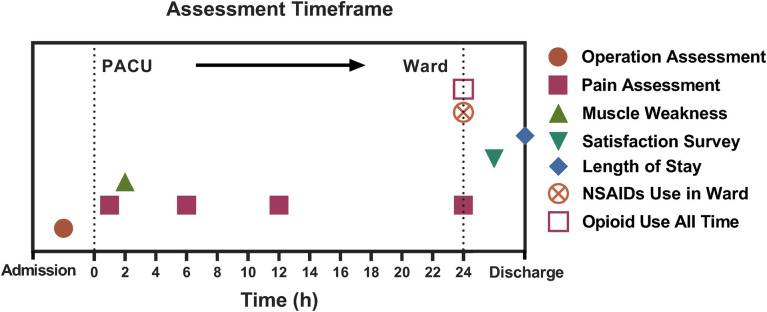
Assessment timeframe for the outcome.

Primary outcome was the postoperative FLACC scale measured at 12 h postoperatively. Secondary outcomes were FLACC scale measured at 1 h, 6 h and 24 h postoperatively. They also included postoperative thumb and shoulder strength, opioid use, NSAIDs use, length of hospital stay, patient satisfaction, surgeon satisfaction, operation time and puncture channels. Assessment timeframe for the outcome can be found in Figure 4.

All the patients were assessed within the 24 h after the operation (1 h, 6 h, 12 h, 24 h). Pain assessment was performed by the anesthetic nurse. The analgesic effect of different groups was assessed by FLACC scale. FLACC scale is a behavioral pain assessment scale specially for nonverbal or preverbal patients. The assessment mainly consist of observation of 5 categories including face, legs, activity, cry, and consolability. The detailed scale could be found in Table 1.

**Table 1:** Details for FLACC scale.

Scale	0	1	2
Face	No particular expression, smile	Occasional grimace or frown, withdrawn disinterested	Frequent to constant frown, clenched jaw, quivering chin
Legs	Normal position, relax	Uneasy, restless, tense	Kicking or legs drawn up
Activity	Lying quietly, normal position, moves easily	Squirming, shifting back/forth, tense	Arched, rigid or jerking
Cry	No cry (Awake or asleep)	Moans, whimpers, occasional complaint	Crying steadily, screams or sobs, frequent complaints
Consolability	Content, relaxed	Reassured by occasional touching, hugging, or talking to distractible	Difficult to console or comfort

The muscle weakness was mainly determined by Oxford muscle strength grading [intact (5/5), reduced (1–4/5), and absent (0/5)] 2 h after the operation. Muscle strength assessment involves recording the extension of the thumb, while shoulder muscle strength is documented for abduction of the shoulder joint. The satisfaction levels of both surgeons and parents were categorized on a scale of satisfied, ambivalent and unsatisfied.

### 2.6 Statistical analysis

All continuous variables were presented as mean ± standard deviation (SD), while ordinal variables were reported as median with interquartile range (IQR). The normality of continuous variables was assessed using the *F*-test, and homogeneity of variances was evaluated with Levene's test. In this study, all continuous variables satisfied the assumptions of normal distribution and homoscedasticity. For continuous variables, Student's *T*-test was used to compare groups. Chi-square test of independence is used for categorical variables. Rank variables were tested by Mann–Whitney *U*-test. The results were considered significant when a *P* value was less than 0.05. All statistics were performed using SPSS 19.0 software.

## 3 Results

### 3.1 Patient basic information analysis

From January 2023 to December 2023, 105 pediatric patients with Gartland Type III SHF were enrolled (Table 2). There were 52 patients (31 males and 21 females) receiving ultrasound-guided coracobrachialis plane musculocutaneous nerve block (Cora-MNB), average age: 4.88 (range: 1–11). Fifty-three patients (34 males and 19 females) were analgecized with supraclavicular brachial plexus block (SC-BPB), average age: 4.91 (range: 1–14). No patients lost follow-up or dropped out of the study. Notably, we observed no significant differences in age or gender across all groups (Table 2). This indicates that the groups were comparable in terms of these demographic factors.

**Table 2:** Basic information of the patients enrolled.

Basic information	Cora-MNB (*n* = 52)	SC-BPB (*n* = 53)	*P*-value
Gender			
Male	31	34	0.632
Female	21	19	
Age	4.88 ± 2.18	4.91 ± 2.44	0.963

### 3.2 Primary outcome

The Cora-MNB group and the SC-BPB group exhibited a significant statistical difference in FLACC scores at 12 h postoperatively (Table 3). The median FLACC score in the Cora-MNB group was 3.0, IQR: 2.0–4.0, whereas the SC-BPB group had a median FLACC score of 5 (3.0–5.0). It indicates that the Cora-MNB group demonstrated superior analgesic effects 12 h after surgery. The FLACC score in the SC-BPB group rebounded, suggesting the analgesic effect of the brachial plexus nerve block had waned.

**Table 3:** Detailed information of FLACC scale [median (IQR)].

Time point	Cora-MNB (*n* = 52)	SC-BPB (*n* = 53)	*P*-value
Post-OP 1 h	2.00 (1.75)	3.00 (2.00)	0.130
Post-OP 6 h	2.00 (2.00)	1.00 (1.50)	0.042 (*)
Post-OP 12 h	3.00 (2.00)	5.00 (2.00)	<0.001 (***)
Post-OP 24 h	1.00 (1.00)	2.00 (1.00)	0.288

*P < 0.05, ***P < 0.001.

### 3.3 Secondary outcomes

Next, we delved into the operational distinctions between ultrasound-guided coracobrachialis plane musculocutaneous nerve block (Cora-MNB) and supraclavicular brachial plexus block (SC-BPB). Our findings revealed that Cora-MNB required considerably less puncture time when compared to the SC-BPB group (Table 4). Furthermore, the number of puncture channels needed for Cora-MNB was significantly lower than that for SC-BPB (Table 5). Ultrasound-guided coracobrachialis plane musculocutaneous nerve block demonstrated a simpler procedure compared to supraclavicular brachial plexus block.

**Table 4:** The detailed information for puncture operation time.

Cora-MNB	SC-BPB	*T*-value	95% CI	*P*-value
4.54 ± 1.21 (min)	9.02 ± 1.94 (min)	14.32	3.88–5.12	<0.001 (***)

***P < 0.001.

**Table 5:** Number of puncture channels.

**Puncture channels**	Cora-MNB (*n* = 52)	SC-BPB (*n* = 53)
1 Channel	45	7
2 Channels	7	30
3 Channels	0	16
Median (IQR)	1.00 (0.00)	2.00 (1.00)
*P*-value	<0.001(*)	

* *p* < 0.001.

Encouragingly, the analgesic effects of Cora-MNB were found to be comparable to those of SC-BPB 1 h and 24 h after the operation (Table 3). In contrast, the Cora-MNB group exhibited consistent and stable analgesic effects compared to perineural injection, possibly attributed to the low diffusion and comprehensive filling of anesthetics around the musculocutaneous nerve or the higher mean dosage on the musculocutaneous nerve (Table 3). Despite a slightly higher FLACC score in Cora-MNB 6 h after the operation, both the groups control the score below 2 and showed effective analgesic effect (Table 3).

We further conducted a comprehensive assessment of opioid and NSAIDs used during both intraoperative and postoperative period. Regardless of intraoperative, postoperative, or cumulative amounts, there was no significant difference in opioid usage between Cora-MNB and SC-BPB (Table 6). However, noteworthy is the substantial reduction in the use of additional non-steroidal anti-inflammatory drugs (NSAIDs) with Cora-MNB, where the pain relieve probability was only 26.4% (14/53) compared to that of SC-BPB (Table 7).

**Table 6:** Detailed information of intraoperative opioid dosage (μg/Kg).

Opioid Dosage (μg/kg)	Cora-MNB	SC-BPB	*T*-value	95% CI	*P*-value
Intraoperative	1.50 ± 0.61	1.45 ± 0.61	0.40	−0.28–0.19	0.692
Postoperative	0.12 ± 0.61	0.25 ± 0.43	1.63	−0.03–0.29	0.106
Total	1.62 ± 0.69	1.70 ± 0.78	0.58	−0.20–0.37	0.565

**Table 7:** Detailed information of NSAIDs aids.

NSAIDs aids	Cora-MNB (*n* = 52)	SC-BPB (*n* = 53)
0 Time	31	14
1 Times	19	37
2 Times	2	2
Median (IQR)	0.00 (1.00)	1.00 (1.00)
*P*-value	0.0014 (**)	

**P < 0.01.

Due to the proximity of the deep fascia of the brachioradialis muscle to the radial nerve, we attempted regional anesthesia injection into the superficial fascia. Practical experience has shown that Cora-MNB has minimal impact on the functionality of the radial nerve. We observed a significant reduction in thumb extensor weakness in Cora-MNB compared to SC-BPB. Only 13.5% (7/45) patients suffered from thumb after extensor weakness coracobrachialis plain musculocutaneous nerve block. However, about 84.9% (45/53) patients have problems in thumb extension after supraclavicular brachial plexus block. As there was no impact on the axillary nerve and suprascapular nerve, Cora-MNB demonstrated a significant preservation of shoulder joint functionality compared to SC-BPB (Table 8).

**Table 8:** Comparison on muscle strength between coracobrachial plain myocutaneous nerve block and supraclavicular brachial plexus block.

Muscle strength	Grade	Cora-MNB (*n* = 52)	SC-BPB (*n* = 53)
Thumb extension strength	Intact	45	8
	Reduced	7	30
	Absent	0	15
	*P*-value	<0.001 (***)	
Shoulder abduction strength	Intact	51	11
	Reduced	1	39
	Absent	0	3
	*P*-value	<0.001 (***)	

***P < 0.001.

**Figure 5:**
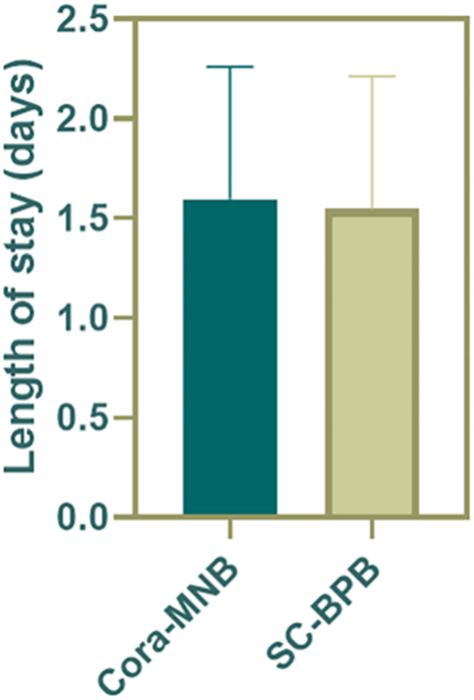
Length of stay in hospital.

Both surgeons and parents expressed approval and satisfaction for the coracobrachialis plain musculocutaneous nerve block (Cora-MNB). In contrast to the 20.7% (11/53) dissatisfaction among surgeons and 16.7% (9/53) dissatisfaction among parents with SC-BPB, Cora-MNB demonstrated a clear and absolute advantage (Table 9). Compared to SC-BPB, Cora-MNB does not increase the length of stay in hospital (Figure 5).

**Table 9:** Patient outcome questionnaires.

Satisfaction	Grade	Cora-MNB (*n* = 52)	SC-BPB (*n* = 53)
Surgical satisfaction	Satisfied	44	12
	Ambivalent	8	30
	Unsatisfied	0	11
	*P*-value	<0.001 (***)	
Parents satisfaction	Satisfied	47	33
	Ambivalent	4	11
	Unsatisfied	1	9
	*P*-value	0.023 (**)	

**P < 0.01, ***P < 0.001.

In summary, our study demonstrates that Cora-MNB is a safe and effective approach for anesthesia in SHF cases.

## 4 Discussion

Supracondylar Humerus Fractures (SHF) is the most common elbow injury in children. It was once referred to as “misconceived fractures” in the 1850s due to its association with skeletal deformities and Volkmann's contracture. In 1959, Gartland introduced a straightforward classification system for straight type SHF. Based on the extent of fracture displacement, SHF were categorized into three types: Type I, with no displacement; Type II, with mild displacement; and Type III, with severe displacement (12).

Severe displacement results in irritation of the periosteum, muscles, and skin, leading to the most intense pain in Gartland Type III SHF. Managing pain for Gartland Type III SHF remains a complex challenge due to complete displacement and the high risk of vascular and nerve injuries. Previous studies have explored various approaches, including local hematoma block (13), Acetaminophen (14), and Ketorolac (15), but there's still limited exploration of anesthesia methods for SHF surgery. In this study, we delve deeper into the impact of different anesthesia techniques on pain management during SHF surgery, with a particular focus on ultrasound-guided coracobrachialis plane musculocutaneous nerve block.

The musculocutaneous nerve is a branch of the brachial plexus. Arising from the anterior roots of C5–C7 and lateral cord, it courses along from the deep to the superficial surface of the coracobrachialis, supplying branches to the biceps and brachialis. The musculocutaneous nerve further descends from the elbow joint to give rise to the lateral antebrachial cutaneous nerve in the forearm. Notably, it also provides innervation to the periosteum of the distal anterior supracondylar region of the humerus (9), which is significant in the context of injury and surgical intervention for SHF. Given the close proximity of the musculocutaneous nerve to the radial nerve within the deep fascial plane, the superficial fascia of the coracobrachialis muscle was finally selected as the plane. Therefore, a musculocutaneous nerve block at coracobrachialis plane might prove to be an effective anesthesia method for treating SHF.

In the current study, both musculocutaneous nerve block and supraclavicular brachial plexus block exhibit similar analgesic effects in the first 6 h postoperatively. However, musculocutaneous nerve block at the coracobrachialis plane provides longer-lasting pain relief. The key factor contributing to the difference is the targeted local administration, which can more effectively block the target nerves, leading to superior pain relief (16). More importantly, different from traditional nerve block approach, our original and novel approach is based on fascial plane nerve blockade, which minimizes excessive absorption and diffusion of the anesthetic, resulting in a prolonged drug retention time and more sustained analgesic effects (17, 18). These improvements were notably reflected in lower FLACC scores 12 h post-surgery.

Previous studies have indicated that the analgesic efficacy of non-steroidal anti-inflammatory drugs (NSAIDs) is not inferior to opioid medications during the perioperative period (19, 20). Therefore, in this study, priority was given to the postoperative use of NSAIDs, and Patient-Controlled Intravenous Analgesia (PCIA) devices were not employed. Despite no significant difference in opioid dosage, more NSAIDs aids were provided to the patient who received supraclavicular brachial plexus block, further demonstrate the advantage of musculocutaneous nerve block.

The variance in blocking levels contributes to differences in potential adverse reactions. Despite the mature ultrasound guidance technology, higher block levels are generally considered more challenging and riskier to perform. On the one hand, there is still a risk of damage to vessels and the pleura in SC-BPB. On the other hand, block of the median nerve, axillary nerve and radial nerve contributes little to analgesia for SHF. However, the coracobrachialis plane musculocutaneous nerve block, employing diffusion within the muscle space, avoids direct needle contact with the nerve, thereby minimizing the potential interference with nerve function. More importantly, it almost perfectly covered the painful area of the SHF. Considering that the periosteal injury in extension-type supracondylar humeral fractures primarily occurs on the anterior aspect of the distal humerus, and that most pediatric patients experience pain in the anterior humerus, it is noteworthy that the musculocutaneous nerve innervates precisely this region, including the periosteum of the distal anterior humerus and surrounding muscle areas (21). Additionally, unlike brachial plexus anesthesia in adults, which is typically administered with the patient awake, pediatric SHF patients undergo the procedure under general anesthesia. This distinction raises concerns about an elevated risk of nerve injury or disruption in children. Fortunately, musculocutaneous nerve block offers a solution to circumvent these issues. Furthermore, the loss of proprioception throughout the arm is an additional drawback of brachial plexus block, which can heighten anxiety and discomfort in pediatric patients, especially in elderly children. Even after the use of lower concentrations of local anesthetics, there are still cases of proprioceptive loss (Three in the current study).

In terms of operational aspects, ultrasound-guided coracobrachialis plane musculocutaneous nerve block can be performed after laryngeal mask placement under general anesthesia, positioning the affected limb in a salute position. In contrast, supraclavicular brachial plexus block requires turning the patient's head to the healthy side and raising the shoulder, increasing operational time and airway management complexity. If an in-plane needle insertion technique is employed for supraclavicular brachial plexus block, the operator must be positioned on the child's side, sometimes requiring head movement to maintain visibility of the needle and probe. However, for musculocutaneous nerve block, the operator was at the head side of the child, allowing for a clearer view of the needle and probe with minimal adjustment, reducing puncture path damage. Importantly, the puncture procedure carries inherent trauma risks, including potential bleeding (22).

The coracobrachialis plane musculocutaneous nerve block offers a distinct advantage in preserving motor function in the shoulder and fingers when compared to the supraclavicular brachial plexus block. This characteristic is particularly beneficial for children, facilitating ultra-early rehabilitation and reducing the risk of postoperative complications (23). Furthermore, considering surgical evaluation, there is a notable concern regarding the potential for radial and median nerve injuries during the surgical procedure. Previous research has demonstrated that longer operative durations and the necessity for open reduction significantly increase the likelihood of nerve palsies (24). Consequently, it becomes crucial for surgeons to assess nerve function post-surgery through finger movements. The supraclavicular brachial plexus block may potentially interfere with this assessment, underscoring the importance of careful consideration.

Both surgeons and parents expressed satisfaction with Cora-MNB. Surgeons were particularly satisfied due to shorter procedure times, improved operative conditions, and enhanced patient pain control. More importantly, Cora-MNB has minimal impact on finger movement compared to brachial plexus anesthesia, allowing surgeons to assess postoperative function without the risk of misjudging intraoperative nerve damage. Furthermore, Cora-MNB does not affect the surgeon's differential diagnosis of compartment syndrome. Parents highly appreciated the analgesic effectiveness of Cora-MNB. Due to the minimal impact of Cora-BNB on hand functionality, children can achieve normal daily life at an early stage, which is also a significant factor contributing to parental satisfaction.

This study has several limitations, including a relatively small sample size, neglecting the varying effects of straight and flexion SHF on patient pain, and the lack of subgroup analysis. Furthermore, due to the specialty of pediatric patients, the study did not assess anesthesia level to statistically measure the success rate of the surgical procedures. Another limitation arises from the postoperative immobilization of the elbow and wrist joints with casts, preventing the evaluation of functionality in these specific joints.

## 5 Conclusion

This study aimed to investigate the effects of different anesthesia methods on postoperative pain management in Gartland Type III Supracondylar Humerus Fractures (SHF) surgery, with a particular focus on the ultrasound-guided coracobrachialis plane musculocutaneous nerve block. Most importantly, musculocutaneous nerve block provided longer-lasting pain relief, likely due to denser fascia tissue, delaying drug absorption and maintaining a high concentration over an extended period. Compared to supraclavicular brachial plexus block, we found that ultrasound-guided coracobrachialis plane musculocutaneous nerve block demonstrated significant advantages, notably the minimal impact on upper limb function and easy operation process. Furthermore, ultrasound-guided coracobrachialis plane musculocutaneous nerve block has gained more satisfaction from both parents and surgeons compared to supraclavicular brachial plexus block.

In conclusion, our study provides valuable insights into the anesthesia methods for Gartland Type III SHF, highlighting the effectiveness and safety of ultrasound-guided coracobrachialis plane musculocutaneous nerve block as a promising option for improved pain management during SHF surgery.

## Data Availability

The data that support the findings of this study are available from the corresponding author upon reasonable request.
